# Redox and autonomic responses to acute exercise-post recovery following *Opuntia ficus-indica* juice intake in physically active women

**DOI:** 10.1186/s12970-021-00444-2

**Published:** 2021-06-07

**Authors:** Marianna Bellafiore, Anna Maria Pintaudi, Ewan Thomas, Luisa Tesoriere, Antonino Bianco, Angelo Cataldo, Dario Cerasola, Marcello Traina, Maria Antonia Livrea, Antonio Palma

**Affiliations:** 1 Sport and Exercise Sciences Research Unit, Department of Psychology, Educational Science and Human Movement, University of Palermo, Via Francesco Spallitta, 52, 90141 Palermo, Italy; 2 STEBICEF Department, Palermo University, 90123 Palermo, Italy

**Keywords:** Oxidative stress, Hydroperoxides, Total antioxidant capacity, Redox balance, Cactus pear juice supplementation, Low frequency, High frequency, Autonomic nervous system

## Abstract

**Background:**

The aim of this study was to investigate if the supplementation with *Opuntia ficus-indica* (OFI) juice may affect plasma redox balance and heart rate variability (HRV) parameters following a maximal effort test, in young physically active women.

**Methods:**

A randomized, double blind, placebo controlled and crossover study comprising eight women (23.25 ± 2.95 years, 54.13 ± 9.05 kg, 157.75 ± 0.66 cm and BMI of 21.69 ± 0.66 kg/m2) was carried out. A juice containing OFI diluted in water and a Placebo solution were supplied (170 ml; OFI = 50 ml of OFI juice + 120 ml of water; Placebo = 170 ml beverage without Vitamin C and indicaxanthin). Participants consumed the OFI juice or Placebo beverage every day for 3 days, before performing a maximal cycle ergometer test, and for 2 consecutive days after the test. Plasma hydroperoxides and total antioxidant capacity (PAT), Skin Carotenoid Score (SCS) and HRV variables (LF, HF, LF/HF and rMSSD) were recorded at different time points.

**Results:**

The OFI group showed significantly lower levels of hydroperoxides compared to the Placebo group in pre-test, post-test and 48-h post-test. PAT values of the OFI group significantly increased compared to those of the Placebo group in pre-test and 48-h post-test. SCS did not differ between groups. LF was significantly lower in the OFI group 24-h after the end of the test, whereas rMSSD was significantly higher in the OFI group 48-h post-test.

**Conclusion:**

OFI supplementation decreased the oxidative stress induced by intense exercise and improved autonomic balance in physically active women.

## Background

It is known that physical exercise induces an alteration in redox balance according to type, intensity, duration and energetic demands of the training stimulus [1–3]. Single intensive bouts of aerobic and anaerobic exercise, as well as chronic exercise promote an increase in the production of reactive oxygen species (ROS) [4, 5], which have different effects on the systemic redox homeostasis depending on the oxidative-stress induced adaptation level [6]. Indeed, the vulnerability of the body to exercise-induced oxidative stress is significantly enhanced in sedentary compared to physically active people [6]. However, scientific evidence still needs to clarify if specific outcomes for ROS generation in health and sport performance are present [7–10]. Agreement although exists regarding oxidative modifications associated to a ROS overproduction, which can limit muscle contraction and induce neuromuscular fatigue, impairing sarcolemmal function, calcium regulation, myofilament interaction and mitochondrial metabolism [8]. However, physiological ROS formation during moderate exercise has an important hormetic function in modulating cell signaling, including the upregulation of antioxidant enzymes [7].

Recently, an increasing number of studies has demonstrated that consumption of specific foods, rich in antioxidants, can ameliorate post-exercise muscle recovery and reduce oxidant load which helps maintaining long-term health in athletes [10, 11]. Among these foods, Opuntia ficus indica (OFI) fruits are considered a valuable ingredient for sports and energy drinks due to the high mineral and vitamin content of calcium, magnesium, potassium, vitamin C [12] and amino acids as proline, glutamine and taurine [13]. Moreover, the characteristic betalains of the OFI fruit have been reported to have health-promoting properties [14, 15]. It has been shown that the consumption of fresh OFI fruit significantly decreases oxidative stress and inflammatory markers, and is associated with an improved antioxidant status in healthy subjects [16, 17]. An example may be found in a study by Khouloud et al. [18], that provided evidence of a reduced exercise-related oxidative stress and muscle damage and improved aerobic performance after 2 weeks of OFI supplementation. These findings suggest that OFI supplementation may be used to improve recovery function following exercise.

A valuable tool employed to measure recovery after exercise, is heart rate variability (HRV) [19]. Different parameters of HRV reflect changes in autonomic nervous system activity and can be altered by high training loads, therefore this tool can be used to evaluate states of fatigue [20].

To the best of our knowledge, there are no studies investigating the effect of OFI supplementation on oxidant and antioxidant production and autonomic status in post-exercise recovery. Therefore, this study aims to investigate the effects of OFI juice supplementation on redox balance and HRV, after a maximal aerobic effort test in physically active women.

## Methods

### Participants

Ten healthy female subjects were recruited for this study by the sporting center of Palermo university (CUS Palermo). Informed consent was mandatory to participate to this study. The institutional Review Board of Palermo University approved the research protocol (Comitato Etico Palermo 1, N. 04, 2018) and all procedures were carried out in accordance with the Declaration of Helsinki (2000). Before data collection, the recruited participant had to fill an anamnesis questionnaire to exclude smokers, those taking medications (including contraceptive pills) or nutritional supplements, or had recent musculoskeletal injures. Moreover, the participants had to report if they were amenorrhoeic. During the first assessment procedures, the participants had to be in the luteal phase of their menstrual cycle, since this seems to correspond to the lowest physiological ROS production period in women [21]. All participants were considered active. Each participant regularly practiced resistance training 3 to 4 times per week. Of the ten subjects who began the study, two did not complete it and were excluded from the final analysis. The characteristics of the remaining eight participants were 23.25 ± 2.95 years of age, 54.13 ± 9.05 kg, 157.75 ± 0.66 cm with a BMI of 21.69 ± 0.66 kg/m^2^.

### Study design

**Fig. 1:**
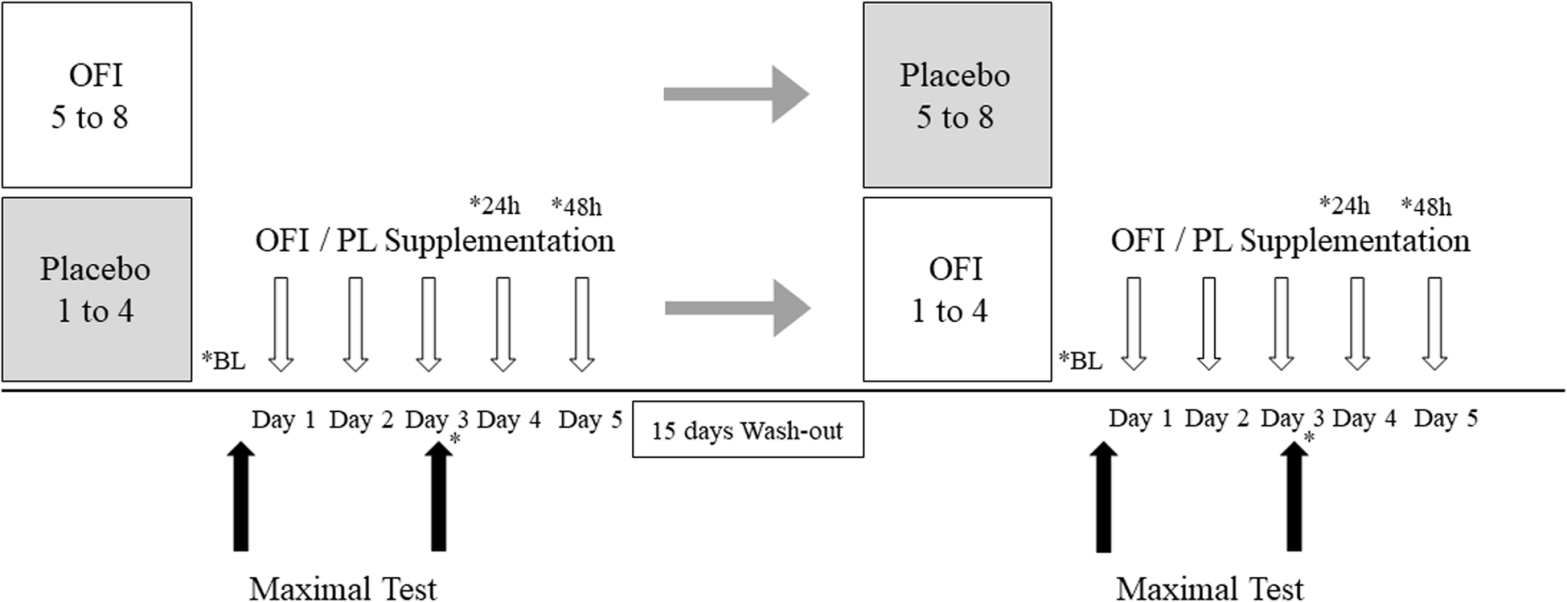
Study design. The subjects were randomly divided into 2 groups and randomly assigned to either a supplementation of 50 ml of concentred peeled fresh fruit juice of Sicilian OFI, which was diluted to 170 ml with water, or 170 ml of Placebo (PL), for 5 days. One week before starting the supplementation, each participant performed the first maximal effort test (BL). The participants consumed OFI or Placebo for 3 days before the second maximal effort test and continued to take it for 2 consecutive days after the testing procedure. After a 2 week wash out period, the treatments were reversed

This is a randomized, double-blind, placebo controlled and crossover design (Fig. 1) study. The participants were randomly divided into two groups, designated as OFI and Placebo. The OFI group received 50 ml of cold-concentrated juice from peeled fresh fruits of Sicilian Opuntia Ficus Indica (NopalRed, Nopal Company srl, Biancavilla, CT, Italy), diluted with water to 170 ml; the Placebo group received 170 ml of a beverage containing the same ingredients of the OFI juice as reported in the commercial preparation, except for the antioxidant Vitamin C and the bioactive phytochemical indicaxanthin. Each participant was instructed to consume either the OFI or Placebo juice along with their breakfast, every day for 3 days before the maximal effort test and continue to take it for 2 consecutive days after the second testing procedure. The supplementation also occurred in the testing days**.** One week before starting the supplementation, all participants were tested (see following sections) in order to determine a baseline measure. In this occasion, covered bottles of OFI or Placebo were distributed to all participants who were instructed about the procedure of supplement intake.

Each variable of interest was collected following specific time points: 1) baseline (BL), 2) pre-test, 3) post-test, 4) 24-h and 5) 48-h after the end of the effort test.

Following a wash out period of 15 days, the supplementation order was reversed and the same procedures as above described, were repeated (Fig. 1). Before the testing sessions, the participants completed a 7-day food diary, from which emerged that their caloric and nutrient ingestion (1881 ± 123 Kcal/die composed of 58% carbohydrate of which 10% sugars, 27% lipids and 15% proteins) was in accordance with the daily intake levels of nutrients recommended for the Italian population [22]. Fruit intake was limited to the OFI juice during the supplementation periods. All participants were invited to follow the same dietary plan 1 week before the testing session in order to limit the influence of nutrition on the analysed parameters.

Plasma hydroperoxides concentration, antioxidant capacity, skin carotenoid score and heart rate variability were collected at baseline (BL), day 3 (pre-test and post-test), day 4 (24 h post-test) and day 5 (48 h post-test) of each period. *Indicates the assessment points.

### Cardio-pulmonary maximal effort test evaluation

All tests were performed between 8 and 10 a.m., after a 12-h overnight fast. Stature and body mass were measured at baseline using a stadiometer and an electronic weighing scale, respectively (SECA, Germany). All participants performed a graded exercise test to exhaustion, on a cycle ergometer. Each participant cycled for 4 min at a constant work rate of 50 W. Thereafter, the cycle ergometer work rate increased by 25 W ∙ min-1 until the participant reached her limit of tolerance. The pedal rate was maintained at 60 rpm throughout the test. A disposable facemask with a 50–70 ml dead space (Cosmed V2, Cosmed Srl, Italy) was used to collect exhaled air throughout the test. Oxygen uptake (VO2) was recorded with a breath-by-breath measurement system (Cosmed Quark CPET, Cosmed Srl, Italy) and maximal oxygen uptake (VO2max) was defined as the highest consecutive 30 s averaged value achieved during the test. The flow meter and gas analysers were calibrated before each test, according to the manufacturer’s instructions. Heart rate was accurately recorded using a short-range radio telemetry system (Polar Electro Oy, Finland). At test cessation an active recovery period was performed at constant lode on the cycle ergometer, pedaling at 25 W at 40 rpm for 3 min, followed by a 2 min passive rest. HR and VO2 were monitored throughout exercise, recovery time and rest. The test was stopped when one or more of the following criteria were met: 1) attainment of a VO2max plateau < 2.2 ml ∙ Kg-1 ∙ min-1; 2) participants were unable to maintain the required work rate.

### Measurement of hydroperoxides concentration and plasma antioxidant capacity

Oxidative stress evaluation was performed with a portable integrated analytical system composed of a photometer and a mini-centrifuge (FRAS 4 Evolvo; H&D Srl, Parma, Italy) as indicated in the study design section. Samples of whole capillary blood were centrifuged for 1:30 min immediately after being collected with a finger puncture, and 10 μL of plasma was used for measuring the concentration of hydroperoxides (using d-ROMs testing) and antioxidant capacity (using PAT testing). The test of active oxygen metabolites (d-ROMs; Diacron International Srl, Grosseto, Italy) measures increases in red color intensity after the addition of a small quantity of plasma to a solution of N,N-diethylparaphenylendiamine (chromogen), buffered to pH 4.8. Such coloring is attributed to the cation radical formation of the amines via oxidation, which is due to alkoxyl and peroxyl radicals. The latter derive from the reaction of the Fe^2+^ and Fe^3+^ ions released by proteins in acidic conditions, as created in vitro. The results are expressed as Units Carratelli (U. CARR), and it has been experimentally established that 1 U. CARR corresponds to 0.08 mg/dL H_2_O_2_. The normal values of a d-ROMs test range from 250 U to 300 U. CARR (i.e., between 20 and 24 mg/dL H_2_O_2_). The plasma antioxidant test (PAT; Diacron International Srl) measures the capacity of a plasma sample to reduce the iron of a colored complex containing ferric ions to its colorless ferrous derivative. The chromatic change of this reaction was photometrically measured at 505 nm, and the results were expressed in U. Cor, in which 1 U. Cor corresponds to 1.4 μmol/L of ascorbic acid used as a standard for reducing the iron. The normal value of a PAT test is > 2200 U. Cor. To maintain consistency, the same set of kits was used for all assessments, and the same operator using the same calibrated machine carried out the evaluations.

### Measurement of skin carotenoid score

Skin carotenoid score (SCS) was measured using a portable device (Pharmanex BioPhotonic Scanner S3, NuSkin, Provo, Utah, USA) based on the Resonance Raman Spectroscopy (RRS). All participants placed the palm of their hand against a light window of the device, twice and held it there for 90 s. During the warm-up process, a black calibration cap was placed over the light window and the scanners self-calibrated using a patented process. When a low intensity laser monochromatic light interacts with some molecules, these diffuse the light emitting a new higher wavelength, monochromatic light that can be revealed by a scanner converting Raman intensity in counts. Because of their conjugated carbon backbone molecular structure, carotenoids possess characteristic vibrational/rotational energy levels that make them particularly well suited for RRS, strongly absorbing in the blue wavelength region and emitting in the green region. The green light emitted from the skin is captured by a highly sensitive light detector. The quantity of dermal carotenoids revealed by RRS can be considered as a marker of the individual antioxidative network and its measurement was applied to assess the body redox state of each participant [23]. Scanner signals are visualized as a coloured scale going red (poor carotenoid score, < 19,000) to dark blue (high carotenoid score, > 50,000). When Raman intensity count difference was  2000 score between scanner scores, participants were scanned a third time. This was done to minimize the individual variation in the scanner score.

### Analysis of heart rate variability and recovery

HRV parameters were collected at baseline, immediately after the maximal graded test, 24 h and 48 h after the end of maximal effort test. Heart rate recovery was measured at 2 min (HRR2) after the end of the test. These measurements were performed with the subjects in a supine position on an examination table for 10 min, during which RR interval recordings were acquired using a portable heart rate monitor (Polar V800, Polar, Finland). The last 5 min of the RR recording were analysed by means of Kubios HRV 2.2 software [24]. HRV time and frequency domains were retrieved [25]. The mean squared differences of successive RR intervals (rMSSD) were retained for the time domain, while the very low frequency (VLF < 0.04 Hz), low frequency (LF from 0.04 to 0.15 Hz) and high frequency (HF from 0.15 to 0.40 Hz) components, in absolute (ms2) and in normalized units (nu) [LFnu: 100 x LF / (total power – VLF), and HFnu: 100 x HF / (total power - VLF)] were retained for the frequency domain. From the values of LF and HF, the LF/HF ratio was determined [26]. The validity of the HRV procedure derived from V800 Polar heart rate monitor is reported elsewhere [27].

### Statistical analysis

Data are reported as means and standard deviations. Normal distribution of the data was verified through the Shapiro-Wilk test. A power analysis was calculated through G*Power v3.1.9.4 (power 0.80; α 0.05; effect size 0.5) estimating a sample of 8 participants. A two-way ANOVA for repeated measures (2 treatments × 4 time-measurements) was used to compare all parameters and identify the source of variation and time-treatment interactions. Sidak’s correction was conducted to compare the OFI and Placebo groups. Furthermore, a one-way within participant’s analysis of variance (ANOVA) was conducted to check for differences in-between times (Pre-test, Post-test, 24 h-post-test, 48 h-post-test). Post-hoc analysis with the Tukey’s test was also perform to detect differences between groups. Effect size using Cohen’s d, for time, treatment and time x treatment interactions have been also reported. Level of statistical significance was set at a *p*-value < 0.05. All analyses were performed with Jamovi (The jamovi project (2021). jamovi (Version 1.6) [Computer Software]. Retrieved from https://www.jamovi.org).

## Results

HR and VO2 measured at rest were 83.25 ± 6.45 bpm and 7.66 ± 2.95 ml/min/Kg, respectively, with no difference between the Placebo and OFI group (*p* > 0.05). Moreover, we did not find any significant difference in VO2max, HRmax and maximal power output reached following the maximal effort test among Placebo, OFI group and baseline conditions (Table 1). Comparing Placebo and OFI groups, we also detected no significant difference in HRR2 after the maximal effort test (Placebo vs OFI: 35.25 ± 3.81 vs. 32.75 ± 4.10 bpm, *p* > 0.05).

**Table 1:** Physiological parameters measured during the cycle ergometer test

	Baseline	Placebo	OFI
VO_2max_ (ml/min/kg)	33.96 ± 2.23	33.41 ± 3.29	35.84 ± 1.84
HR_max_ (bpm)	177.13 ± 8.43	178.00 ± 9.64	176.88 ± 8.90
Power (Watt)	143.75 ± 17.68	143.75 ± 17.68	150.00 ± 18.90

*VO*_*2max*_ Maximal Oxygen Consuption, *HR*_*max*_ Maximal Heart Rate

**Fig. 2:**
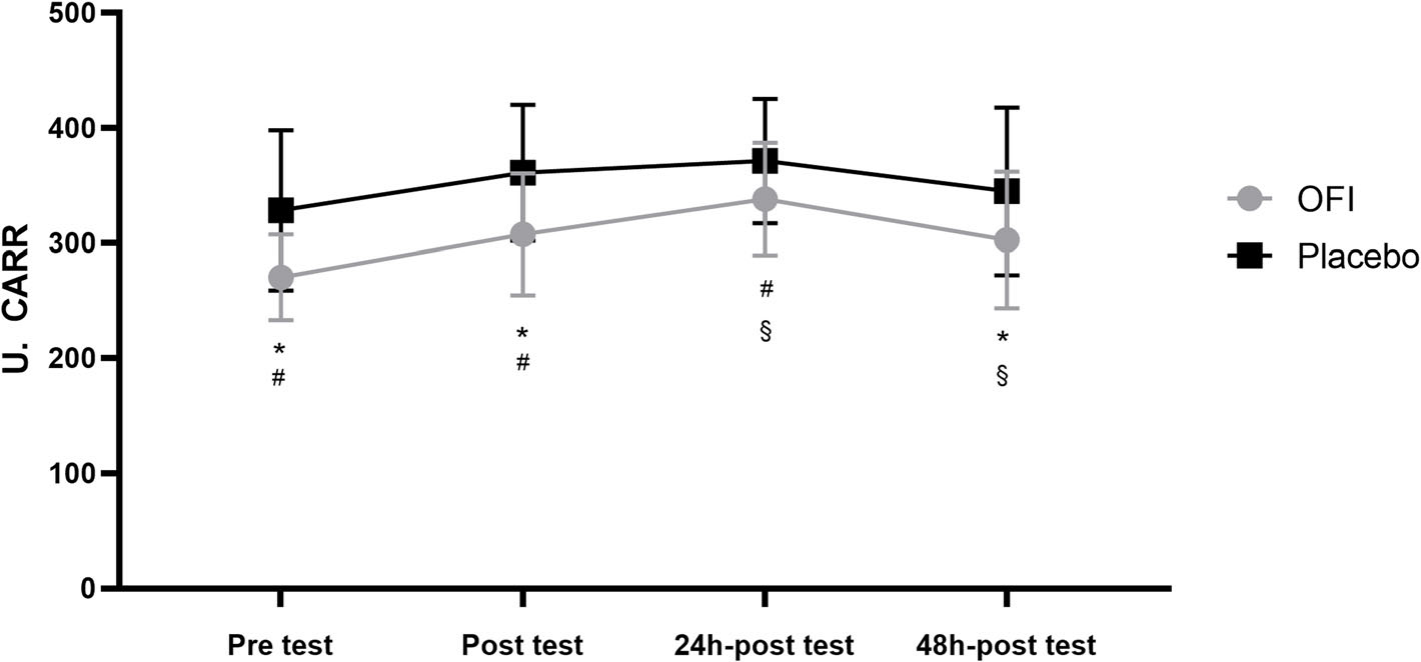
Changes in plasma hydroperoxide levels in response to OFI or PL supplementation before and immediately, 24 h and 48 h after a maximal effort test. *p < 0.05 OFI pre-, post- and 48 h post-test vs. corresponding PL groups; #p < 0.05 OFI pre-test vs post-test and 24 h post-test; §p < 0.05 OFI 24 h post-test vs 48 h post-test

ANOVA showed a significant effect of the treatment (F = 86.11; *p* < 0.0001; d = 0.27) and the time (F = 6.43; *p* = 0.0029; d = 0.81) but no interaction between these factors (F = 0.154; *p* = 0.57; d = 0.01) for hydroperoxides levels. We observed lower hydroperoxide concentrations in OFI compared to the Placebo group in all the examined conditions (Fig. 2). In detail, OFI showed a significant decrease in pre-test (*p* = 0.0013), post-test (*p* = 0.0030) and 48 h-post-test (*p* = 0.0230) compared with the corresponding conditions of the Placebo group (Fig. 2). Baseline hydroperoxides’ concentration was not significantly different compared with Placebo and OFI levels in pre and post-test conditions (*p* > 0.05). In the OFI group (Fig. 2), oxidant concentration significantly increased immediately after the test (*p* = 0.0212) and 24 h after the test completion compared with the pre-test levels (*p* = 0.0056), while it significantly decreased after 48 h-post-test respect to 24 h-post-test (*p* = 0.0136). Conversely, statistical analysis did not display any significant difference within the Placebo group.

**Fig. 3:**
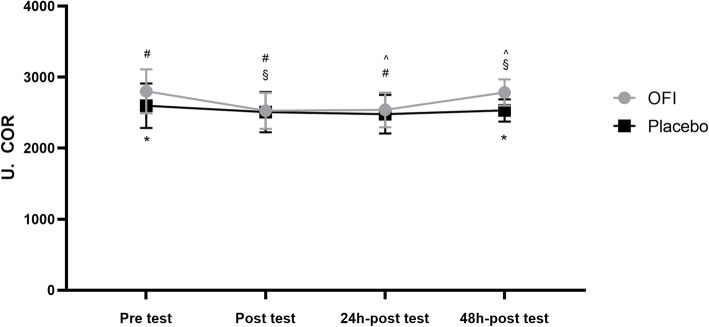
Measurement of plasma antioxidant capacity following OFI or PL supplementation before,immediately after, 24 h and 48 h after a maximal effort test. *p < 0.05 OFI pre- and 48 h post-test vs corresponding PL groups; #p < 0.05 OFI pre-test vs post-test and 24 h post-test; §p < 0.05 OFI post-test vs 48 h post-test; ˆp < 0.05 OFI 24 h post-test vs 48 h post-test

Concerning PAT, we found that, treatment (F = 11.64; *p* = 0.0113; d = 0.52) but not time (F = 3.648; *p* = 0.089; d = 0.08) interactions, while no interaction between these factors was present (F = 0.746; *p* = 0.09; d = 0.04). As shown in Fig. 3, PAT levels in OFI were significantly higher than Placebo group during pre-test (*p* = 0.0385) and 48 h-post-test (*p* = 0.0072). After 3 days of OFI supplementation, OFI group exhibited a significant higher concentration of PAT compared to baseline condition (*p* = 0.0028). In OFI group, PAT levels significantly declined immediately after the effort test (*p* = 0.0049) and 24 h after the end of the test (p = 0.0072) compared to those before testing (Fig. 3), while they increased 48 h post-test compared to post-test (*p* = 0.0080) and 24 h-post-test (*p* = 0.0115). No significant difference in all the examined conditions was present in the Placebo group (Fig.3).

**Fig. 4:**
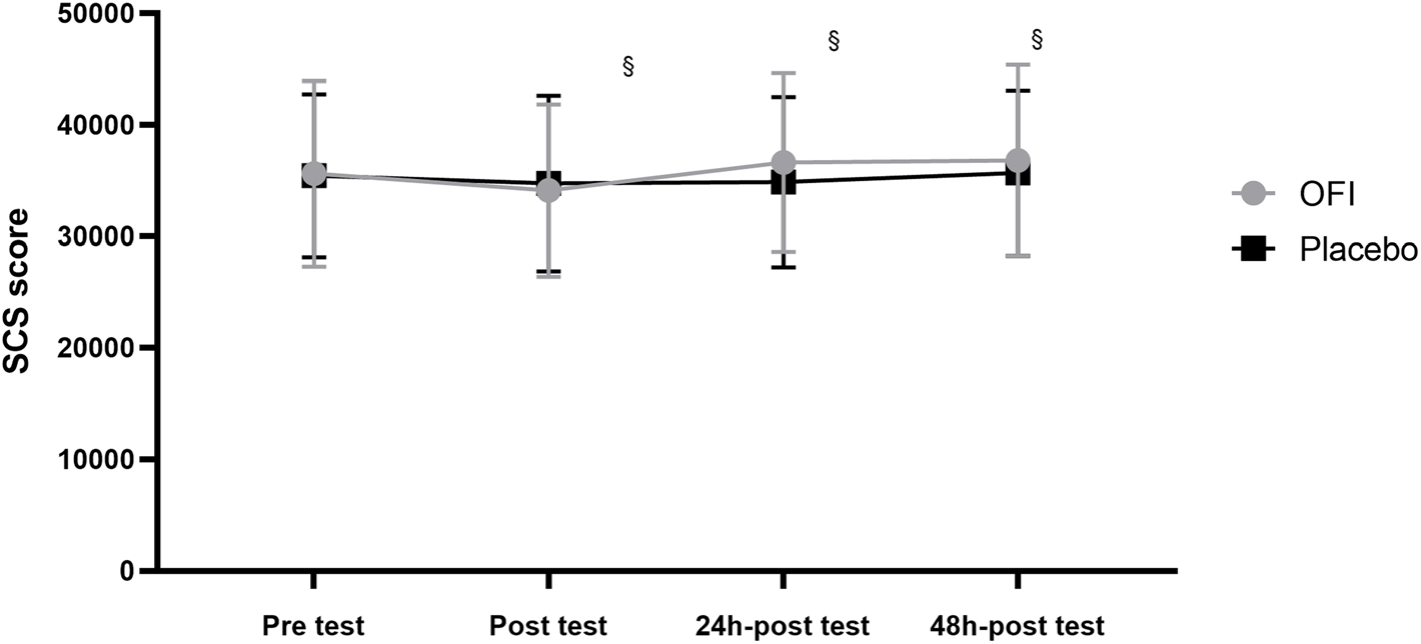
Variations in Skin Carotenoid Score based on the Resonance Raman Spectroscopy following OFI or PL supplementation before, immediately after, 24 h and 48 h after a maximal effort test. §*p* < 0.05 OFI post-test vs 24 h-post-test and 48 h post-test

Statistical analysis revealed a significant effect of time for the SCS score (F = 7.337; *p* = 0.0015; d = 0.52), whereas no significant effect of the treatment (F = 0.6119; *p* = 0.4597; d = 0.07) and the interaction between these factors was detected (F = 0.071; *p* = 0.0920; d = 0.01). No significant difference was highlighted between Placebo and OFI groups (*p* > 0.05) (Fig. 4). OFI group showed a significant increase in SCS score after 24 h (*p* = 0.0063) and 48 h (*p* = 0.0031) of recovery compared with that immediately after the test (Fig. 4). No difference was present in the Placebo group (*p* > 0.05).

Regarding HRV parameters, we found that LF values were significantly dependent on treatment (F = 11.25; *p* = 0.0047; d = 0.87) and time (F = 3.056, *p* = 0.0386; d = 0.39). Moreover, the interaction between these two factors was also significant (F = 3.187, *p* = 0.033; d = 0.69). As reported in the Table 2, LF parameter of the OFI group significantly decreased compared to the Placebo group 24 h after the end of the test (*p* = 0.0007). In OFI group, LF value in 24 h post-test was lower than pre-test (*p* = 0.0309) and post-test (*p* = 0.0012). In the Placebo group, there were no significant differences among the examined conditions (*p* > 0.05). Further, we did not observe any significant effect on HF and LF/HF parameters by treatment and time variables (p > 0.05, Table 2). By contrast, these variables significantly affected rMSSD parameters for treatment (F = 10.57; *p* = 0.0058; d = 1.06) and time (F = 5.338; *p* = 0.0033; d = 0.26), while no interaction was revealed between them (F = 0.464; *p* = 0.61; d = 0.26). As reported in the Table 2, rMSSD value of OFI group was higher than the Placebo group in 48 h post-test (*p* = 0.0117). Moreover, the OFI group showed a decline in post-test respect to pre-test (p0.0249), while an increase was observed at the 48 h post-test compared to the post-test (*p* = 0.0464). Conversely, no difference was present in the Placebo group (*p* > 0.05).

**Table 2:** Changes in HRV between Placebo and OFI group after the cycle ergometer test

	Group	Pre test	Post test	24 h-post test	48 h-post test
LF (ms^2^)	Placebo	66.86 ± 7.09	66.21 ± 8.04	66.91 ± 8.20^*a*^	65.12 ± 3.87
	OFI	62.79 6.04^*c*^	66.90 ± 10.37^*d*^	52.83 ± 4.47^*acd*^	58.11 ± 5.78
HF (ms^2^)	Placebo	33.00 ± 7.03	28.74 ± 2.72	32.98 ± 8.20	33.02 ± 4.46
	OFI	33.38 ± 3.52	34.31 ± 9.31	31.39 ± 4.39	36.81 ± 8.54
LF/HF	Placebo	2.15 ± 0.69	2.32 ± 0.33	2.20 ± 0.83	2.01 ± 0.33
	OFI	1.90 ± 0.26	2.11 ± 0.68	1.71 ± 0.23	1.69 ± 0.58
RMSSD	Placebo	51.68 ± 7.40	42.18 ± 9.64	46.55 ± 9.20	45.53 ± 7.93^*b*^
	OFI	59.40 ± 8.44^*e*^	48.63 ± 6.18^*ef*^	54.85 ± 9.44	58.48 ± 7.84^*bf*^

All data are presented as means±SD. *LF* Low Frequency, *HF* High Frequency, *LF/HF* Low-high frequency ratio; *rMSSD* Root Mean Square of the Successive Differences. *a,b,c,d,e* and *f* denote significant differences (*p* < 0.05) between groups and within the same group

## Discussion

The main objective of our investigation was to examine if the supplementation with OFI juice for a short time could modify redox and autonomic status in healthy active women after a single bout of maximal aerobic exercise. Our results show a reduced production of hydroperoxides and an increased antioxidant capacity in the OFI juice supplemented group compared to the placebo group before the test and 48 h after the end of the test. Moreover, the HRV parameters showed a tendency to increased parasympathetic activity and reduced sympathetic activity, which corresponds to improved recovery function following an intense exercise.

Despite moderate exposure to ROS is necessary to induce adaptive responses to training, high-intensity exercise typically results in overproduction of reactive oxygen species [28]. The overproduction of ROS destroys the cell redox-balance and activates various redox-dependent transcription factors finally inducing formation of inflammatory proteins, activation of inflammatory processes and further ROS formation [29]. Counter wise, moderate intensity exercise has been demonstrated to prevent oxidative stress [30]. Nutrition and nutritional supplements are frequently employed strategies to limit the overproduction of ROS [31]. Being high in nutritional and bioactive phytochemicals [32], OFI has received considerable attention in the scientific community as a potential source of natural antioxidants as Vitamin C and bioactive phytochemicals as betalains [16], and as direct functional food [33]. It is known that OFI consumption positively decreases plasma pro-inflammatory markers and oxidative damage to lipids, while increases plasma anti-inflammatory markers and improves antioxidant status in healthy humans [16, 17]. Conversely, little is known about the effects of OFI ingestion in response to physical exercise.

In a previous investigation, we showed that a 48 h recovery following a high intensity training session was not sufficient to restore redox balance in amateur rhythmic gymnasts [3]. Our recommendation was to contrast the temporary redox imbalance caused by high intensity exercise, consuming a balanced diet rich in natural antioxidants and phytochemicals. To have a scientific evidence, the present randomized, double-blind, placebo-controlled and crossover study assessed the antioxidant capacity of OFI juice to counteract oxidative stress induced by a single bout of maximal exercise in physically active women. We observed that the plasma hydroperoxides significantly increased after testing, however the increase was smaller in the group supplemented with OFI than placebo group, thus suggesting that OFI juice was able to limit the formation of ROS and increase antioxidant capacity. Our previous study [16] and the known activity of indicaxanthin as an antioxidant and modulator of the redox balance of cells under a number of pathophysiological conditions [34, 35] suggest a role for this phytochemical in the observed effect. With respect to OFI supplementation, Khouloud et al.^**18**^ following an intermittent yo-yo maximal aerobic effort test, not only described a decrease of oxidative stress following OFI supplementation, but also a reduction in the total and LDL cholesterol. Other investigation by the same research group [36] has also evaluated the effects of OFI supplementation following a maximal anaerobic test and similar results for oxidative stress and inflammatory markers were retrieved. Therefore, it seems that OFI supplementation, regardless of the typology of exercises proposed, is able to reduce oxidative stress and promote antioxidant responses. However, in our best knowledge few studies have described antioxidant responses of OFI intake following a maximal exercise [18, 36] and no study has examined redox status during the recovery period. Unlike other studies, we did not observe an increased total antioxidant capacity immediately after the maximal exercise [18, 36]. We found, instead, a significantly higher antioxidant capacity after 3 days of supplementation and 48 h after the end of the maximal exercise in the OFI group. These findings suggest that OFI juice ingestion may contribute, with endogenous antioxidants, to decline the levels of ROS and restore redox balance after exercise-induced stressing stimuli.

To examine the body redox balance, we analysed SCS, which represents a biomarker of overall antioxidant status measuring the quantity of dermal carotenoids with RSS analysis [37]. It has been indeed established that skin carotenoids as α-, γ-, β-carotene, lutein, zeaxanthin, lycopene and their isomers are degraded by stress factors and therefore may serve as markers of antioxidative status [38]. Although SCS significantly increased immediately, 24 h and 48 h after the maximal effort test in response to OFI supplementation, similar values were also present after the placebo supplementation. Changes in human carotenoid levels have been observed across seasons [38], however these have not been observed after relatively short periods [39]. Our investigation comprised of a 5-day supplementation period followed by a 15-day washout, a very restricted time frame, which probably did not allow a measurable variation in skin carotenoid concentration.

Regarding the duration of OFI supplementation, we used a short-term intake because it is known that a chronic supplementation could blunt the adaptive effect of exercise training, interfering with the endogenous antioxidant response to ROS and negatively affecting athletic performance and recovery [40]. Improvements in exercise performance have been observed following acute antioxidant supplementation associated with highly fatiguing exercise [36, 41].

There is increasing body of evidence indicating that the consumption of specific foods, high in antioxidant compounds, can decrease post-exercise recovery time [42, 43]. As an example, cherries and blue berries seem to facilitate the recovery of muscular strength following strenuous eccentric exercise [42, 43]. In our study, we assessed the physiological recovery after the maximal effort test by monitoring the changes in the autonomic nervous system patterns, which reflect changes in HRV measures. A recent paper has discussed if HRV may be an effective method of assessing oxidative stress, concluding that reflections in the time and frequency domain are associated to variations in oxidative markers [44]. Increases in HRV and oxidative stress have been also associated to several pathological conditions [45, 46], however modifications in lifestyle and exercise typology may improve such parameters [30, 47]. The most commonly used HRV measures include rMSSD, LF and HF, which are respectively expression of the parasympathetic, sympathetic and parasympathetic activity levels [25]. Our results have identified changes in different HRV parameters between the placebo and OFI group. In particular, a significant increase in rMSSD and a decrease in LF, 24 h and 48 h after the maximal effort test, were retrieved. These results suggest that OFI supplementation contributes to accelerate recovery time following maximal effort exercise and re-stablish autonomic balance.

To the best of our knowledge, only the study of Schmitt and colleagues [48] has evaluated the effects of OFI intake on parameters of HRV, reporting very similar results compared to those here provided. The overall modifications observed in the HRV parameters may suggest vagal activation and restoration of autonomic activity [49].

The main limitation to this study is the limited number of included participants and the short supplementation period. However, the double-blind, cross-over design has allowed us to effectively evaluate the effects of the supplementation. In addition, we should also consider that sleep records were not retained, therefore we cannot determine if sleep deprivation may have influenced our results. OFI juice can be recommended to physically active people who want to contrast exercise-induced oxidative stress and restore autonomic balance induced by high intensity exercise through a balanced diet rich in natural antioxidant supplements.

## Conclusions

The main results of our study suggest that supplementation with a fruit juice of Sicilian Opuntia Ficus Indica (cactus pear) is able to improve redox and autonomic balance in healthy women. These findings may be useful for coaches and people involved in sports nutrition in order to include such nutritional elements in a healthy balanced diet. The inclusion of OFI juice can reduce redox imbalance caused by high intensity exercise and therefore may also be beneficial to avoid overreaching or overtraining in healthy women.

## Data Availability

The datasets used and/or analysed during the current study are available from the corresponding author on reasonable request.

## Abbreviations

ANOVA: analysis of variance
BP: baseline
HRmax: maximal heart rate
HRR2: Heart rate recovery was measured at 2 min
HRV: heart rate variability
OFI: *Opuntia ficus-indica*
PAT: total antioxidant capacity
PL: Placebo
RER: Respiratory Exchange Ratio
rMSSD: mean squared differences of successive RR intervals
ROS: reactive oxygen species
RRS: Resonance Raman Spectroscopy
SCS: Skin Carotenoid Score
U.CARR: Units Carratelli
VO2max: maximal oxygen uptake
